# Recurrent Neoantigens in Colorectal Cancer as Potential Immunotherapy Targets

**DOI:** 10.1155/2020/2861240

**Published:** 2020-07-17

**Authors:** Chao Chen, Songming Liu, Ruokai Qu, Bo Li

**Affiliations:** ^1^BGI-Shenzhen, Shenzhen 518083, China; ^2^BGI Education Center, University of Chinese Academy of Sciences, Shenzhen 518083, China; ^3^BGI-GenoImmune, BGI-Shenzhen, Wuhan 430074, China

## Abstract

This study was aimed at investigating the mutations in colorectal cancer (CRC) for recurrent neoantigen identification. A total of 1779 samples with whole exome sequencing (WES) data were obtained from 7 published CRC cohorts. Common HLA genotypes were used to predict the probability of neoantigens at high-frequency mutants in the dataset. Based on the WES data, we not only obtained the most comprehensive CRC mutation landscape so far but also found 1550 mutations which could be identified in at least 5 patients, including *KRAS* G12D (8%), *KRAS* G12V (5.8%), *PIK3CA* E545K (3.5%), *PIK3CA* H1047R (2.5%), and *BMPR2* N583Tfs∗44 (2.8%). These mutations can also be recognized by multiple common HLA molecules in Chinese and TCGA cohort as potential “public” neoantigens. Many of these mutations also have high mutation rates in metastatic pan-cancers, suggesting their value as therapeutic targets in different cancer types. Overall, our analysis provides recurrent neoantigens as potential cancer immunotherapy targets.

## 1. Introduction

Colorectal cancer (CRC) is the third most common malignancy in the world and the second leading cause of cancer-related mortality [1, 2]. Traditional treatments, such as surgery, chemotherapy, and radiation, have been important in prolonging patients' survival, but for patients with advanced CRC, especially those with metastatic disease, these treatments are limited and often intolerant [3].

In recent years, immunotherapy, including immune checkpoint inhibitors (ICIs), cancer vaccines, and neoantigen-based tumor-infiltrating lymphocytes (TILs), has played an increasingly important role in cancer therapy [4]. Certain CRC patients with high microsatellite instability (MSI-H) could potentially benefit from ICIs treatment [5]. However, not all CRC patients with MSI-H show clinical efficacy in ICI treatment. Neoantigen-based immunotherapy is complementary to ICIs since it has no specific requirement for patient's MSI status nor tumor mutation burden (TMB) [6, 7]. Previous studies on CRC genomics mainly focused on the mechanism of tumor development and metastasis and less involves neoantigen and neoantigen-based immunotherapy [8–13]. By integrating the mutation data of already existing CRC cohorts and combining the common HLA genotypes in these populations [14, 15], our study is expected to find the common neoantigens in CRC patients and facilitate further development of off-the-shelf neoantigen-based immunotherapy.

## 2. Materials and Methods

### 2.1. Genomic Data of CRC

This study was approved by the Institutional Review Board on Bioethics and Biosafety of BGI group. All somatic mutations, including single-nucleotide variants (SNVs) and short insertion/deletion (indels), were downloaded from the latest publications (Table 1 and Supplementary Table S1), which represent seven geographically diverse study groups involving 1779 CRC patients. Since all data used in this study were from public databases with informed consent from participants in the original genome study, no additional informed consent was required.

### 2.2. Pipeline for Neoantigen Prediction

For neoantigen prediction, a total of 43 HLA genotypes were selected with frequencies greater than 5% in the Chinese or TCGA cohort. Mutations present in at least 5 patients, including 476 SNVs and 974 indels, were selected for subsequent neoantigen analysis. A total of five software are used for affinity prediction between neoantigen peptide and HLA alleles, which are NetMHC [16], NetMHCpan [17], PickPocket [18], PSSMHCpan [19], and SMM [20]. Candidate high-affinity peptides were further predicted by EPIC [21]. EPIC is a neoantigen prediction software based on mass spectrometry-derived motifs and tissue-specific expression profiles. It considers various complex factors in antigen presentation process, such as affinity and tumour-specific gene expression, and can accurately predict epitope presentation. If the sample is not quantified for gene expression, the software's default expression value (TPM = 4) is used. According to our previous research [22], neoantigen peptides need to meet the following four criteria: (1) between 8 and 11 meters in length; (2) affinity IC50 < 500 nM in at least two tools; (3) mutant (MT) peptides affinity score lower than the wild type (WT); and (4) the presentation score of EPIC > 0.5.

### 2.3. Statistical Analysis

The statistical analysis was done in R-studio and the mutation analysis and drawing were done with the maftools package [23]. If no special instructions were given, *P* < 0.05 was considered significant.

## 3. Results

### 3.1. The Integrated Mutation Landscape of CRC Patients

**Figure 1:**
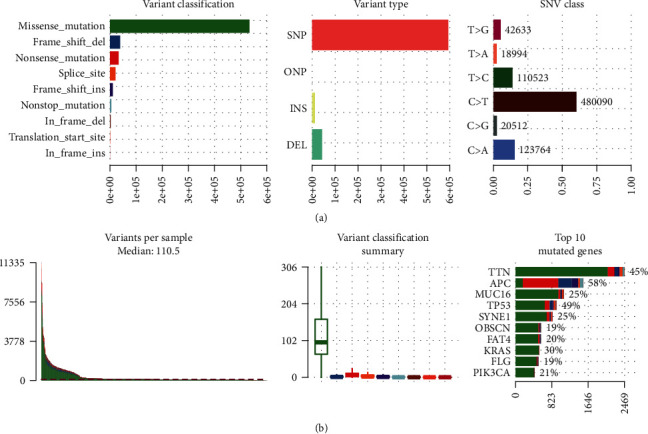
The mutation landscape in the CRC cohort. (a) From left to right, counts of each variant classification, counts of each variant type, and counts of each SNV class. (b) From left to right, variant number per sample, variant classification, and top 10 significantly mutated genes.

The mutation profiles of all CRC samples are shown in Figure 1 and Figure S1. In general, missense mutation is the main type of mutations. At the base substitution level, C>T is the dominant mutant type, followed by C>A (Figure S2), which is consistent with TCGA and previous reports [24, 25]. The median number of mutations in each sample was 110, and *APC*, *MUC16*, *TP53*, *SYNE1*, *KRAS*, and *PIK3CA* were the most frequently mutated genes. Apart from the samples without MSI status, there are 203 MSI-H samples in the combined cohort (Table 1), accounting for 11.4%. The mutation load of MSI-H samples was higher than that of MSS samples (Figure S3-S4). Interestingly, through the integration analysis of the MSS samples, we found the mutation of four genes, including *TENM1*, *SOX9*, *PIK3CA*, and *KRAS*, and the mutation of *TP53* gene were mutually exclusive (Fisher's exact test, *P* < 0.05, Figure S5). This different mutation pattern may suggest that the carcinogenic mechanisms are different in CRC patients carrying mutations in these four genes and in those carrying *TP53* mutation. Correspondingly, there is no such mutual exclusion effect in the MSI-H samples (Figure S6).

There are many hotspot mutations in CRC samples. Of these, 1550 recurrent mutations could be identified in at least 5 patients, with 476 SNVs and 974 indels. Previous studies have shown that common neoantigens in cancers could be used as potential immunotherapy targets [22, 26]. Therefore, in order to find out whether there are common neoantigens in CRC populations, we used these mutations in downstream analyses to predict tumour-specific neoantigens.

### 3.2. Neoantigens Shared among CRC Patients

Due to the difference in the frequency of HLA in different populations, in order to search for “public” neoantigens in CRC populations, we selected high-frequency HLA in Chinese (HLA frequency > 5% in Han Chinese [15]) and high-frequency HLA in TCGA (HLA frequency > 5% in TCGA [27]) for neoantigen analysis. Finally, a total of 43 HLA alleles were used for neoantigen prediction (Table S2).

We detected 274 SNV-derived neoantigens and 1269 indel-derived neoantigens (Table S3-4). Each SNV usually produces 1-2 high-affinity peptides, while each indel can produce multiple high-affinity peptides. The top ten high-frequency SNV and indel-related neoantigens are shown in Tables 2 and 3, respectively. In terms of SNV, mutations of *KRAS*, *PIK3CA*, *PCBP1*, and *CHEK2* can produce 10 neoantigens with the highest frequency (Table 2 and Table S3). In terms of indel, although the mutation frequency is not as high as SNV's, generally one indel can produce about 5-10 neoantigen peptides (Table 3 and Table S4).

### 3.3. Comparison of Neoantigens in Different Subtypes of CRC

**Figure 2:**
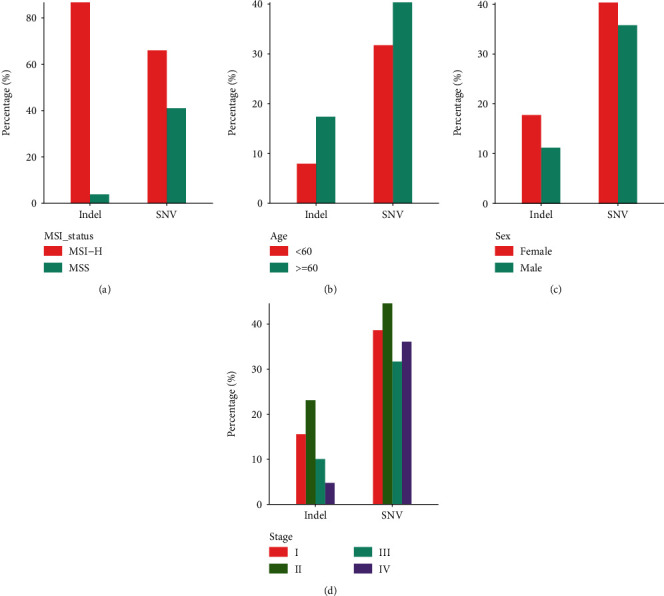
The comparison of neoantigens between different subgroups: (a) between different MSI statuses; (b) between age ≥ 60 and age < 60 groups; (c) between female and male groups; (d) between different stages. These analyses excluded patients with unknown subtypes.

By comparing the neoantigen profiles of different subtypes of CRC, we found that there were more SNV- and indel-derived neoantigens in MSI-H patients than in MSS patients (Fisher's exact test, *P* < 0.01, Figure 2(a)). The 1269 indel-related neoantigens can cover 86.7% of patients with MSI-H CRC, but only 3.9% of MSS patients were covered, indicating that indel is the main source neoantigens of MSI-H CRC. SNV-derived neoantigens can cover 41% of MSS patients and 66% of MSI-H patients.

Patients older than 60 carry more neoantigens than those under 60 years of age (Fisher's exact test, *P* < 0.01, Figure 2(b)). Women tend to carry more neoantigens than men, especially those derived from indels. Female patients accounted for 62.2% in the MSI-H cohort and 51.2% in the MSS cohort. The proportion of MSI-H subtypes was higher in female CRC patients (Fisher's exact test, *P* value = 0.007); this may partly explain why the results show that women have a higher neoantigen load than men. In terms of cancer stage, Stage II CRC patients carry the most abundant neoantigens, which may be related to the mutation load of the corresponding subgroup (Figures 2(c) and 2(d)). We compared the percentage of patients with MSI-H in Stage II and other stages, finding 28% of patients are MSI-H in Stage II while 10.1% in other stages (Fisher's exact test, *P* value < 0.001). We also analyzed the mutated rates of *KRAS* and *PIK3CA* in Stage II CRC patients. For *KRAS*, the mutation rate was 28.8% in Stage II while 28.4% in other stages, without significant difference. For *PIK3CA*, the mutation rate of *PIK3CA* in Stage II patients was higher than that in other stages, with the mutation rate of 25.6% and 17.1%, respectively (Fisher's exact test, *P* value < 0.001). According to the above analysis results, the higher neoantigen load of patients at Stage II may be due to the higher proportion of MSI-H or the higher mutation rate of *PIK3CA* in these patients.

### 3.4. Hotspot Mutation-Related Neoantigens That May Be a Potential Source of Immunotherapy Target in CRC and Pan-Cancer

**Figure 3:**
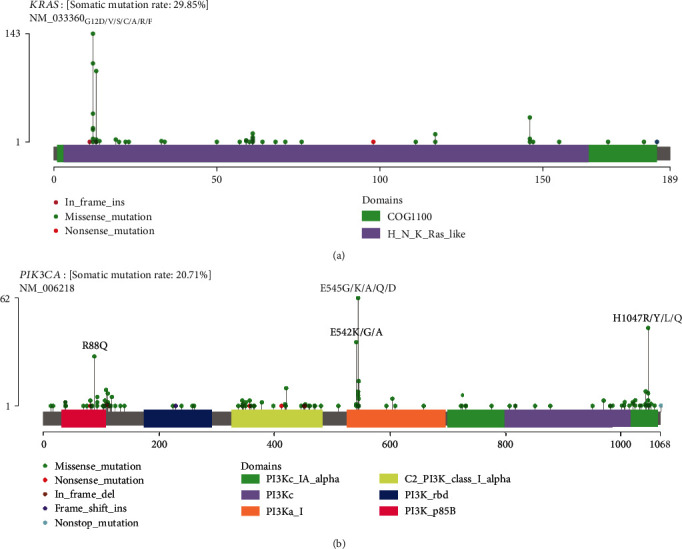
Mutational spectrum of KRAS (a) and PIK3CA (b) in 1179 CRC patients.

To further investigate the potential significance of these high-frequency neoantigens, we focused on mutations with the highest frequency, including *KRAS* G12D, *KRAS* G12V, *PIK3CA* E545K, and *PIK3CA* H1047R, because these mutations not only produce recurrent neoantigens but also have a higher frequency in the CRC cohort (Figures 3(a) and 3(b)).

**Figure 4:**
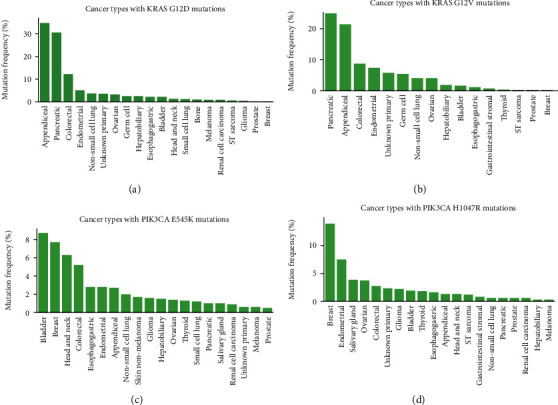
Mutation frequency in MSK-IMPACT cohorts. *KRAS* G12D (a), *KRAS* G12V (b), *PIK3CA* E545K (c), and *PIK3CA* H1047R (d) in MSK-IMPACT pan-cancer cohorts.

*KRAS* Gly12 (including G12V, G12C, and G12D) is a classic driver mutation that occurs more than 20% in the metastatic pancreatic and appendiceal cancers [24]. Both G12D and G12V are highly mutated in multiple metastatic cancers, including endometrial, CRC, and non-small cell lung cancer (Figures 4(a) and 4(b)). Mayakonda et al. have reported the high frequency of *KRAS* G12D mutations in pancreatic cancer [23, 25]. Liang et al. also demonstrated by mass spectrometry in 2019 that *KRAS* G12V-mutated neoantigen can be presented by HLA-A11:01 cell lines [28]. And as far as we know, clinical trials for *KRAS* G12V mutations in patients with HLA-A11:01 are already under way (NCT03190941).

*PIK3CA* is one of the driver genes in gastrointestinal malignancies [29, 30]. *PIK3CA* E545K is a hotspot mutation in breast cancer and has corresponding first-line drugs (Alpelisib and Fulvestrant) [31]. In addition to breast cancer, this mutation could be found in more than 5% bladder, head and neck, and colorectal cancer patients (Figure 4(c)). *PIK3CA* H1047R is most commonly found in breast cancer [32] and is also frequently mutated among multiple tumor types in the MSK-IMPACT metastatic cancers (Figure 4(d)). Our previous work has shown that the epitopes of this mutation can be presented by multiple HLA molecules (e.g., HLA-C07:02, HLA-C 07:01, HLA-A30:01, and HLA-B58:01) and can be a potential neoantigen in patients with gastric cancer [22]. Combined with the results of this study, it is suggested that this mutation can be used as an important therapeutic target for patients with gastrointestinal tumors.

## 4. Discussion

There are many different types of antigens that can be used as targets for immunotherapy, such as tumor-associated antigens, cancer-testis antigens, and neoantigens [33]. Tumor-associated antigens (e.g., ERBB2/HER2) are highly expressed in tumor and poorly expressed in normal tissues, so they may serve as therapeutic targets for some tumor types, but the disadvantage is that they may cause nonspecific immune responses. Cancer-testis antigens (e.g., NY-ESO-1 antigen [34] and MAGE [35]) are not expressed in normal adult cells except in reproductive tissues (e.g., testis, fetal ovaries, and trophoblast cells). However, both tumor-associated antigens and cancer-testis antigens are prone to severe immune responses. For example, attempts to target melanoma-associated antigen 3 (MAGE-A3) with adoptive cell therapy have resulted in severe neurotoxicity and death, which may be related to the expression in the brain of MAGE-A family members that has not been previously recognized [36]. Compared with the above two antigen types, neoantigen has stronger immunogenicity and tumor specificity, so it is an ideal target for immunotherapy [37, 38]. However, due to the difference of neoantigen among patients, the current neoantigen-based immunotherapy is completely individualized. Since the human exome region is about 30 M, the probability of the same mutation between different individuals is relatively low, and the epitope of the mutation is presented by specific HLA allele, so the possibility of the same neoantigen epitope between different individuals is very low.

However, not all mutations in tumors are random. Current genomic studies have shown that there are many hotspot mutations in driver genes, and the neoantigen epitopes formed by these mutations are potential “public” immunotherapy targets, such as *KRAS* G12D/V and *CDK4* R24C/L [39]. In CRC, the frequency of frameshift mutations is higher in patients with MSI-H subtype, and it has been reported that frameshift peptides have been used in the clinical treatment of CRC (NCT01461148). In our study, we also found that indel-related neoantigens can cover a large proportion of MSI-H CRC patients. At present, 10-20 neoantigen peptides are usually synthesized at the same time to prepare vaccines or other immunotherapy products [40, 41]. If combined with the neoantigens corresponding to hotspot point mutations and frameshift mutations, it is believed that these neoantigen can cover more CRC patients, so that more patients can benefit from neoantigen-based immunotherapy.

## 5. Conclusion

Based on the analysis of the published WES data of CRC, the most complete mutation landscape of CRC was obtained. We selected HLA subtypes with high frequency in Chinese and TCGA cohort to predict the common neoantigens in the population. The high-frequency mutations, including *KRAS* G12D (8%), *KRAS* G12V (5.8%), *PIK3CA* E545K (3.5%), *PIK3CA* H1047R (2.5%), and *BMPR2* N583Tfs∗44 (2.8%), can be recognized and presented by many HLA genotypes, such as HLA-A1101, HLA-A03:01, and HLA-B57:01. These HLA genotypes are the main HLA subtypes in Chinese and Americans, indicating the broad spectrum of the neoantigens we identified. In conclusion, we have found a series of “public” neoantigens for CRC, which provide important resources for immunotherapy of CRC in the future.

## Figures and Tables

**Table 1:** Summary of clinical information of CRC cohort, including patients from seven studies.

Characteristic	Baylor	Beijing	COCA-CN	Genetech	Harvard	TCGA	Texas	Total
	(*n* = 110)	(*n* = 98)	(*n* = 321)	(*n* = 74)	(*n* = 619)	(*n* = 528)	(*n* = 29)	(*n* = 1779)
Age (years)								
<60	38 (34.5%)	48 (49.0%)	155 (48.3%)	0 (0%)	67 (10.8%)	158 (29.9%)	0 (0%)	466 (26.2%)
≥60	70 (63.6%)	50 (51.0%)	166 (51.7%)	0 (0%)	550 (88.9%)	366 (69.3%)	0 (0%)	1202 (67.6%)
Unknown	2 (1.8%)	0 (0%)	0 (0%)	74 (100%)	2 (0.3%)	4 (0.8%)	29 (100%)	111 (6.2%)
Sex								
Female	65 (59.1%)	50 (51.0%)	127 (39.6%)	0 (0%)	380 (61.4%)	253 (47.9%)	15 (51.7%)	890 (50.0%)
Male	45 (40.9%)	48 (49.0%)	194 (60.4%)	0 (0%)	239 (38.6%)	273 (51.7%)	14 (48.3%)	813 (45.7%)
Unknown	0 (0%)	0 (0%)	0 (0%)	74 (100%)	0 (0%)	2 (0.4%)	0 (0%)	76 (4.3%)
Stage								
I	12 (10.9%)	10 (10.2%)	40 (12.5%)	0 (0%)	152 (24.6%)	94 (17.8%)	0 (0%)	308 (17.3%)
II	42 (38.2%)	44 (44.9%)	94 (29.3%)	0 (0%)	187 (30.2%)	196 (37.1%)	0 (0%)	563 (31.6%)
III	48 (43.6%)	39 (39.8%)	130 (40.5%)	0 (0%)	159 (25.7%)	150 (28.4%)	1 (3.4%)	527 (29.6%)
IV	8 (7.3%)	4 (4.1%)	56 (17.4%)	0 (0%)	65 (10.5%)	69 (13.1%)	28 (96.6%)	230 (12.9%)
Unknown	0 (0%)	1 (1.0%)	1 (0.3%)	74 (100%)	56 (9.0%)	19 (3.6%)	0 (0%)	151 (8.5%)
MSI status								
MSI-H	24 (21.8%)	8 (8.2%)	0 (0%)	15 (20.3%)	91 (14.7%)	65 (12.3%)	0 (0%)	203 (11.4%)
MSI-L	0 (0%)	0 (0%)	0 (0%)	0 (0%)	0 (0%)	77 (14.6%)	0 (0%)	77 (4.3%)
MSS	81 (73.6%)	32 (32.7%)	0 (0%)	59 (79.7%)	438 (70.8%)	346 (65.5%)	29 (100%)	985 (55.4%)
Unknown	5 (4.5%)	58 (59.2%)	321 (100%)	0 (0%)	90 (14.5%)	40 (7.6%)	0 (0%)	514 (28.9%)

**Table 2:** Top ten SNVs and the corresponding neoantigens in the CRC cohort.

Chr	Location	Gene	AA change	Peptide	Frequency	HLA types
chr12	25398284	*KRAS*	G12D	VVVGADGVGK	143	A11:01
chr12	25398284	*KRAS*	G12V	VVGAVGVGK	104	A11:01
chr3	1.79E+08	*PIK3CA*	E545K	STRDPLSEITK	63	A03:01; A11:01
chr3	1.79E+08	*PIK3CA*	E545K	ITKQEKDFLW	63	B57:01
chr3	1.79E+08	*PIK3CA*	H1047R	ARHGGWTTK	45	B27:05
chr3	1.79E+08	*PIK3CA*	R88Q	REEFFDETRQL	30	B40:01
chr2	70315174	*PCBP1*	L100Q	RPPVTQRLVV	28	B07:02
chr2	70315174	*PCBP1*	L100Q	SRPPVTQRL	28	C06:02; C07:01; C07:02
chr22	29091840	*CHEK2*	K373E	SEILGETSL	21	B18:01; B40:01
chr12	25398284	*KRAS*	G12A	VVVGAAGVGK	19	A11:01

**Table 3:** Top ten indels and the corresponding frequency in the CRC cohort.

Chr	Location	Gene	AA change	Frequency
chr2	203420130	*BMPR2*	N583Tfs∗44	50
chr10	890939	*LARP4B*	T163Hfs∗47	37
chr1	1290110	*MXRA8*	R301Gfs∗107	31
chr18	34205516	*FHOD3*	S336Vfs∗138	29
chr15	45003781	*B2M*	L15Ffs∗41	28
chr12	110019240	*MVK*	A141Rfs∗18	27
chr3	168833257	*MECOM*	G614Efs∗30	26
chr22	20130522	*ZDHHC8*	T459Rfs∗177	20
chr8	103289349	*UBR5*	E2121Kfs∗28	20
chr6	158508009	*SYNJ2*	P1113Lfs∗5	19

## Data Availability

Data supporting the results of this study are available from corresponding authors on request.

## Supplementary Materials

Supplementary Materials Figure S1: the landscape of 25 significantly mutated genes in all the CRC patients. Figure S2: ratio of base conversion and transversion in mutations. Figure S3: the landscape of 25 significantly mutated genes in MSI-H CRC patients. Figure S4: the landscape of 25 significantly mutated genes in MSS CRC patients. Figure S5: somatic interaction analysis among the significantly mutated genes found four genes mutually exclusive with TP53 in MSS samples. Figure S6: somatic interaction analysis in MSI-H samples. Table S1: the list of clinical information of samples in previous seven studies of CRC. Table S2: the list of high-frequency HLA genotypes in Chinese and TCGA. Table S3: the list of SNV derived neoantigens. Table S4: the list of indel-derived neoantigens.
