# Supplementary material for: Recurrent Neoantigens in Colorectal Cancer as Potential Immunotherapy Targets
Source: Biomed Res Int. 2020 Jul 17;2020:2861240. doi: 10.1155/2020/2861240 (PMC7383341; doi:10.1155/2020/2861240)
Supplement: Supplementary Materials — Figure S1: the landscape of 25 significantly mutated genes in all the CRC patients. Figure S2: ratio of base conversion and transversion in mutations. Figure S3: the landscape of 25 significantly mutated genes in MSI-H CRC patients. Figure S4: the landscape of 25 significantly mutated genes in MSS CRC patients. Figure S5: somatic interaction analysis among the significantly mutated genes found four genes mutually exclusive with TP53 in MSS samples. Figure S6: somatic interaction analysis in MSI-H samples. Table S1: the list of clinical information of samples in previous seven studies of CRC. Table S2: the list of high-frequency HLA genotypes in Chinese and TCGA. Table S3: the list of SNV derived neoantigens. Table S4: the list of indel-derived neoantigens. [file 2861240.f1.zip › Figure S1.pdf]

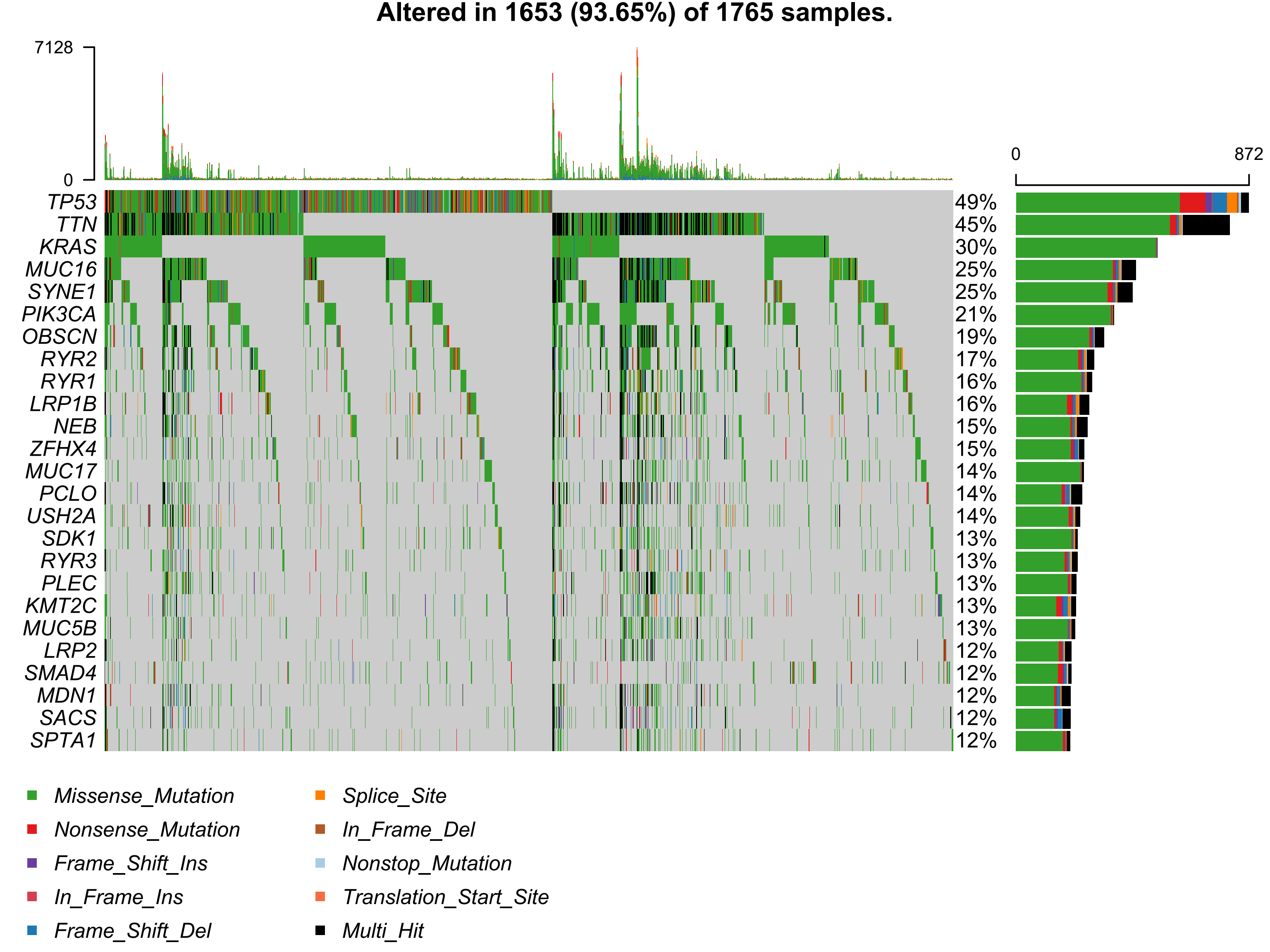

Altered in 1653 (93.65%) of 1765 samples.
7128
0
0
872
TP53 49%
TTN 45%
KRAS 30%
MUC16 25%
SYNE1 25%
PIK3CA 21%
OBSCN 19%
RYR2 17%
RYR1 16%
LRP1B 16%
NEB 15%
ZFHX4 15%
MUC17 14%
PCLO 14%
USH2A 14%
SDK1 13%
RYR3 13%
PLEC 13%
KMT2C 13%
MUC5B 13%
LRP2 12%
SMAD4 12%
MDN1 12%
SACS 12%
SPTA1 12%
Missense_Mutation
Nonsense_Mutation
Frame_Shift_Ins
In_Frame_Ins
Frame_Shift_Del
Splice_Site
In_Frame_Del
Nonstop_Mutation
Translation_Start_Site
Multi_Hit
