# Supplementary figures and images for: Recurrent Neoantigens in Colorectal Cancer as Potential Immunotherapy Targets
Source: Biomed Res Int. 2020 Jul 17;2020:2861240. doi: 10.1155/2020/2861240 (PMC7383341; doi:10.1155/2020/2861240)

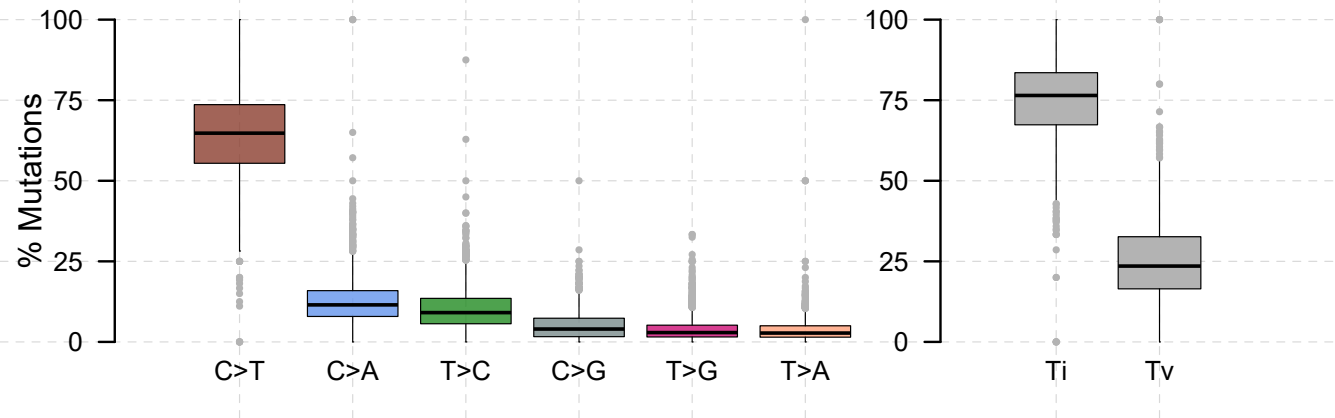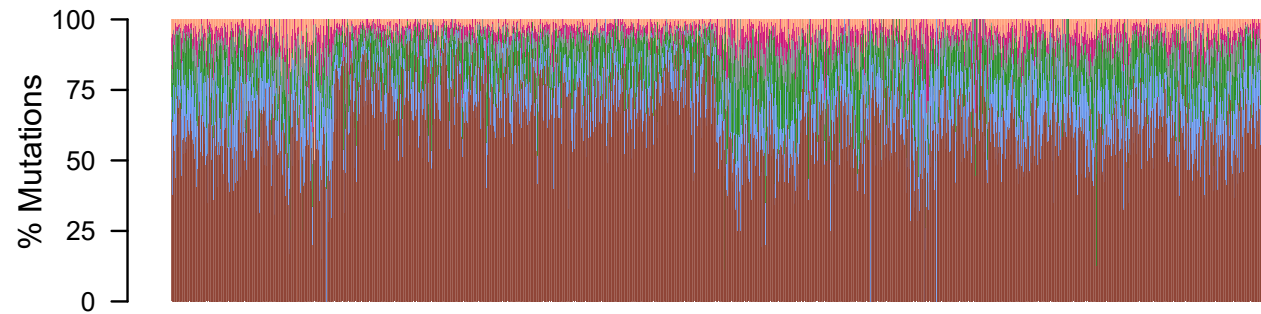

Supplement: Supplementary Materials — Figure S1: the landscape of 25 significantly mutated genes in all the CRC patients. Figure S2: ratio of base conversion and transversion in mutations. Figure S3: the landscape of 25 significantly mutated genes in MSI-H CRC patients. Figure S4: the landscape of 25 significantly mutated genes in MSS CRC patients. Figure S5: somatic interaction analysis among the significantly mutated genes found four genes mutually exclusive with TP53 in MSS samples. Figure S6: somatic interaction analysis in MSI-H samples. Table S1: the list of clinical information of samples in previous seven studies of CRC. Table S2: the list of high-frequency HLA genotypes in Chinese and TCGA. Table S3: the list of SNV derived neoantigens. Table S4: the list of indel-derived neoantigens. [file 2861240.f1.zip › Figure S2.pdf]

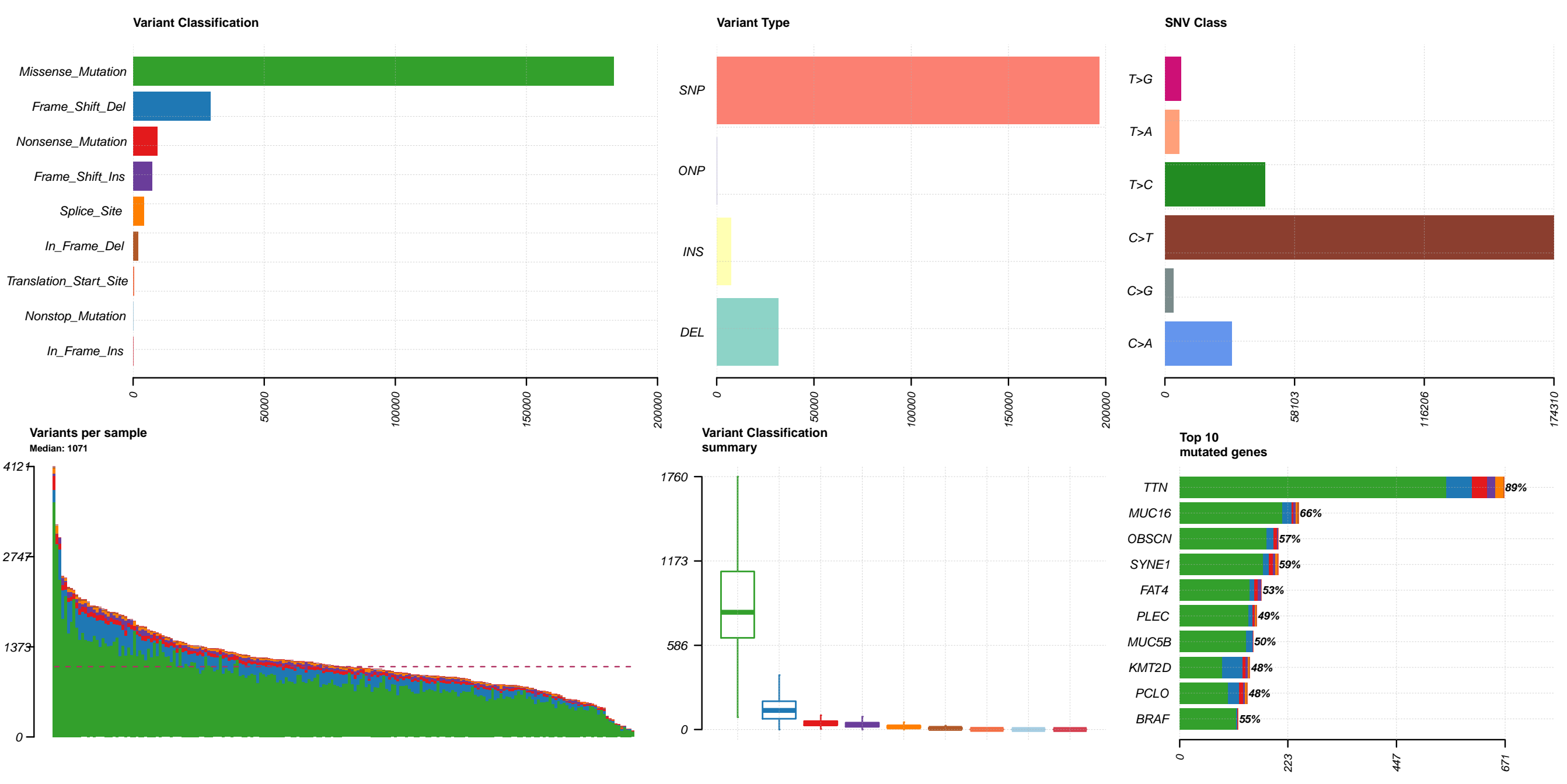

Supplement: Supplementary Materials — Figure S1: the landscape of 25 significantly mutated genes in all the CRC patients. Figure S2: ratio of base conversion and transversion in mutations. Figure S3: the landscape of 25 significantly mutated genes in MSI-H CRC patients. Figure S4: the landscape of 25 significantly mutated genes in MSS CRC patients. Figure S5: somatic interaction analysis among the significantly mutated genes found four genes mutually exclusive with TP53 in MSS samples. Figure S6: somatic interaction analysis in MSI-H samples. Table S1: the list of clinical information of samples in previous seven studies of CRC. Table S2: the list of high-frequency HLA genotypes in Chinese and TCGA. Table S3: the list of SNV derived neoantigens. Table S4: the list of indel-derived neoantigens. [file 2861240.f1.zip › Figure S3.pdf]

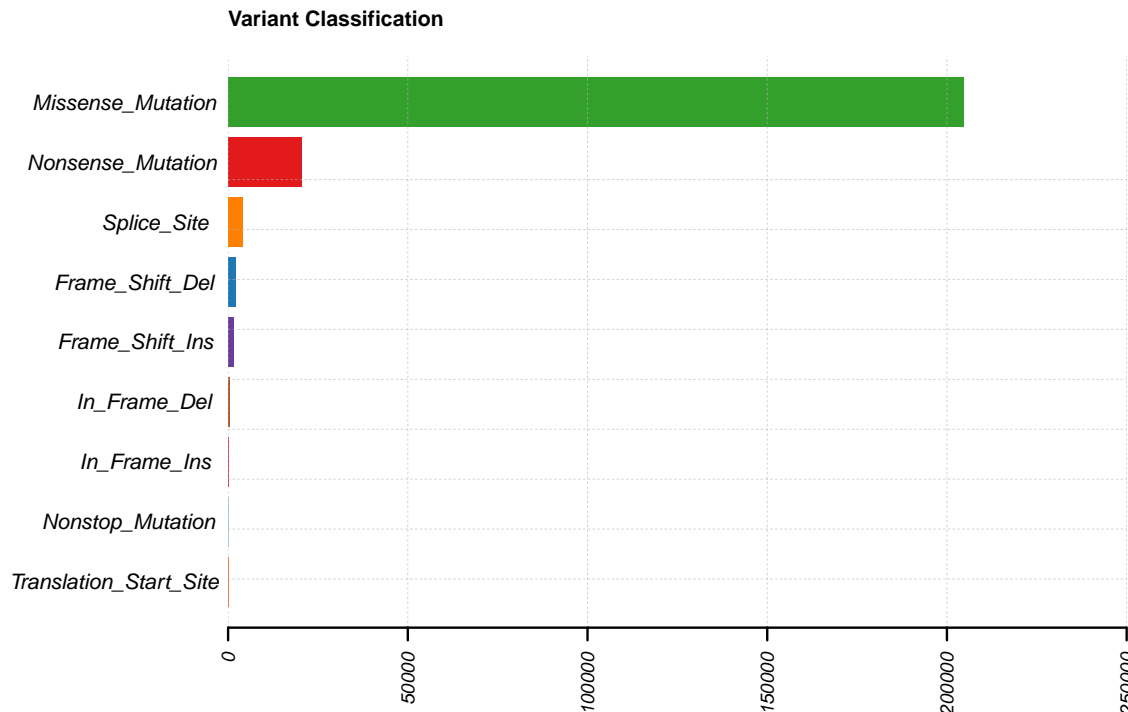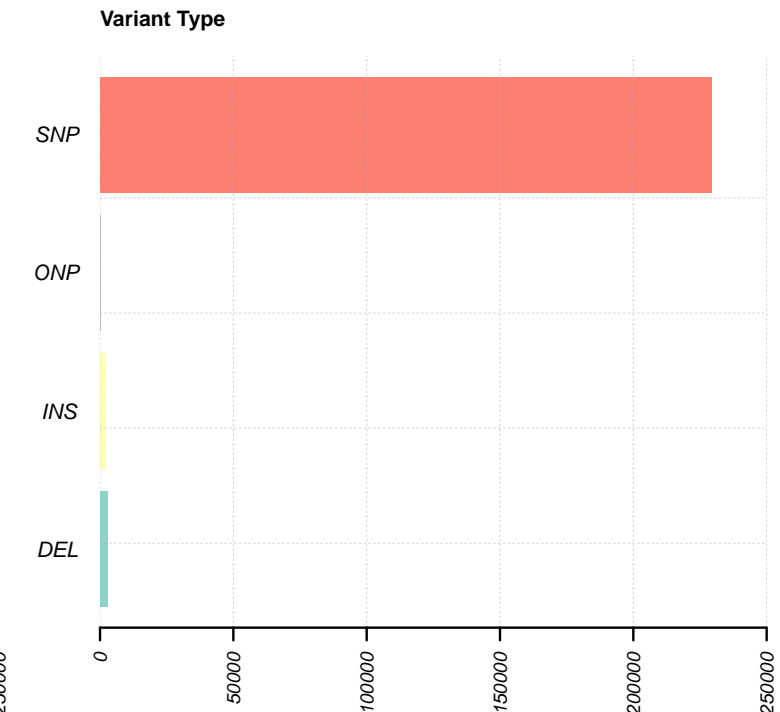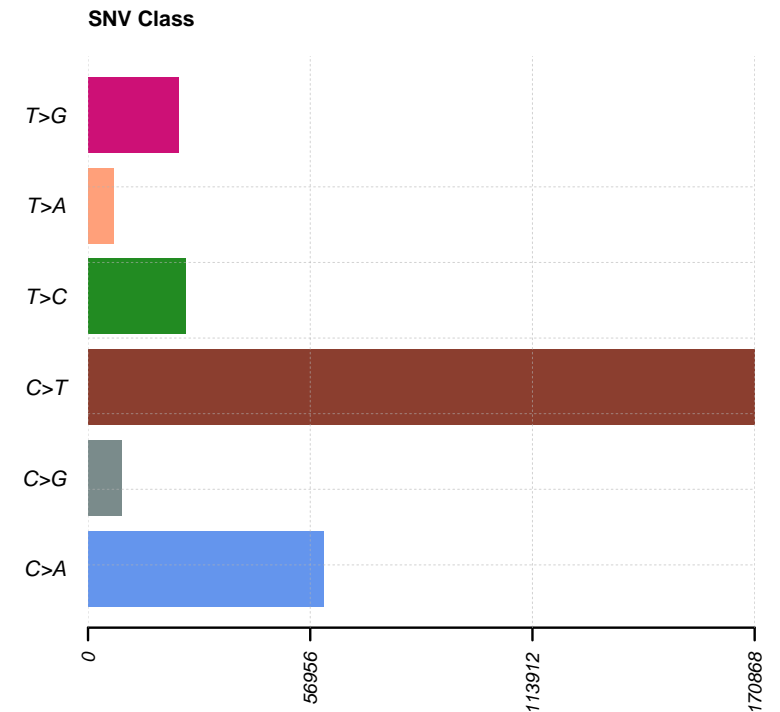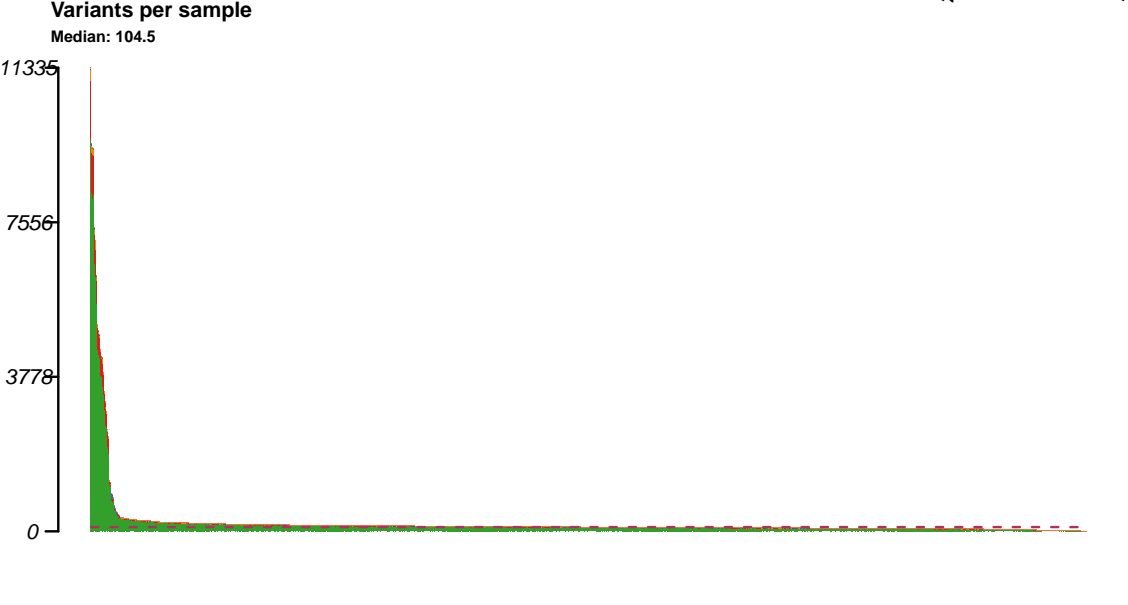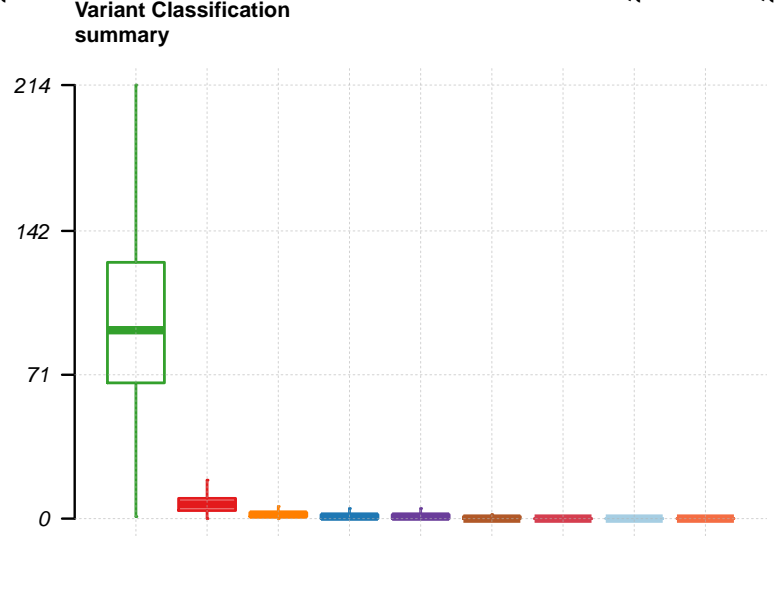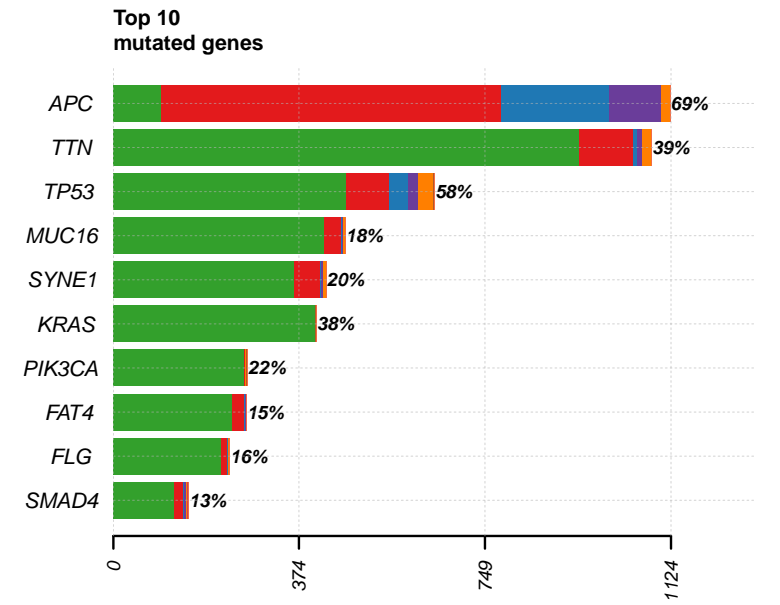

Supplement: Supplementary Materials — Figure S1: the landscape of 25 significantly mutated genes in all the CRC patients. Figure S2: ratio of base conversion and transversion in mutations. Figure S3: the landscape of 25 significantly mutated genes in MSI-H CRC patients. Figure S4: the landscape of 25 significantly mutated genes in MSS CRC patients. Figure S5: somatic interaction analysis among the significantly mutated genes found four genes mutually exclusive with TP53 in MSS samples. Figure S6: somatic interaction analysis in MSI-H samples. Table S1: the list of clinical information of samples in previous seven studies of CRC. Table S2: the list of high-frequency HLA genotypes in Chinese and TCGA. Table S3: the list of SNV derived neoantigens. Table S4: the list of indel-derived neoantigens. [file 2861240.f1.zip › Figure S4.pdf]

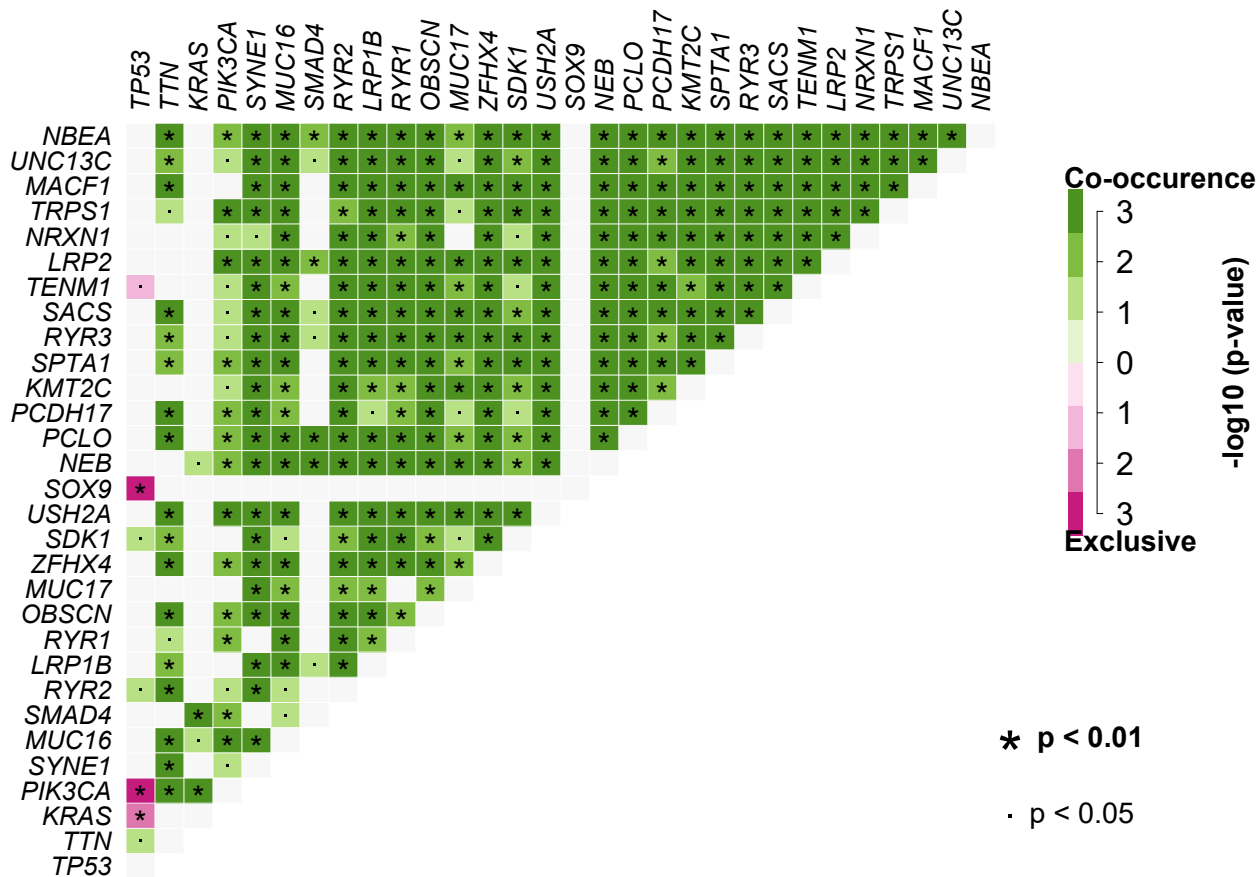

Supplement: Supplementary Materials — Figure S1: the landscape of 25 significantly mutated genes in all the CRC patients. Figure S2: ratio of base conversion and transversion in mutations. Figure S3: the landscape of 25 significantly mutated genes in MSI-H CRC patients. Figure S4: the landscape of 25 significantly mutated genes in MSS CRC patients. Figure S5: somatic interaction analysis among the significantly mutated genes found four genes mutually exclusive with TP53 in MSS samples. Figure S6: somatic interaction analysis in MSI-H samples. Table S1: the list of clinical information of samples in previous seven studies of CRC. Table S2: the list of high-frequency HLA genotypes in Chinese and TCGA. Table S3: the list of SNV derived neoantigens. Table S4: the list of indel-derived neoantigens. [file 2861240.f1.zip › Figure S5.pdf]

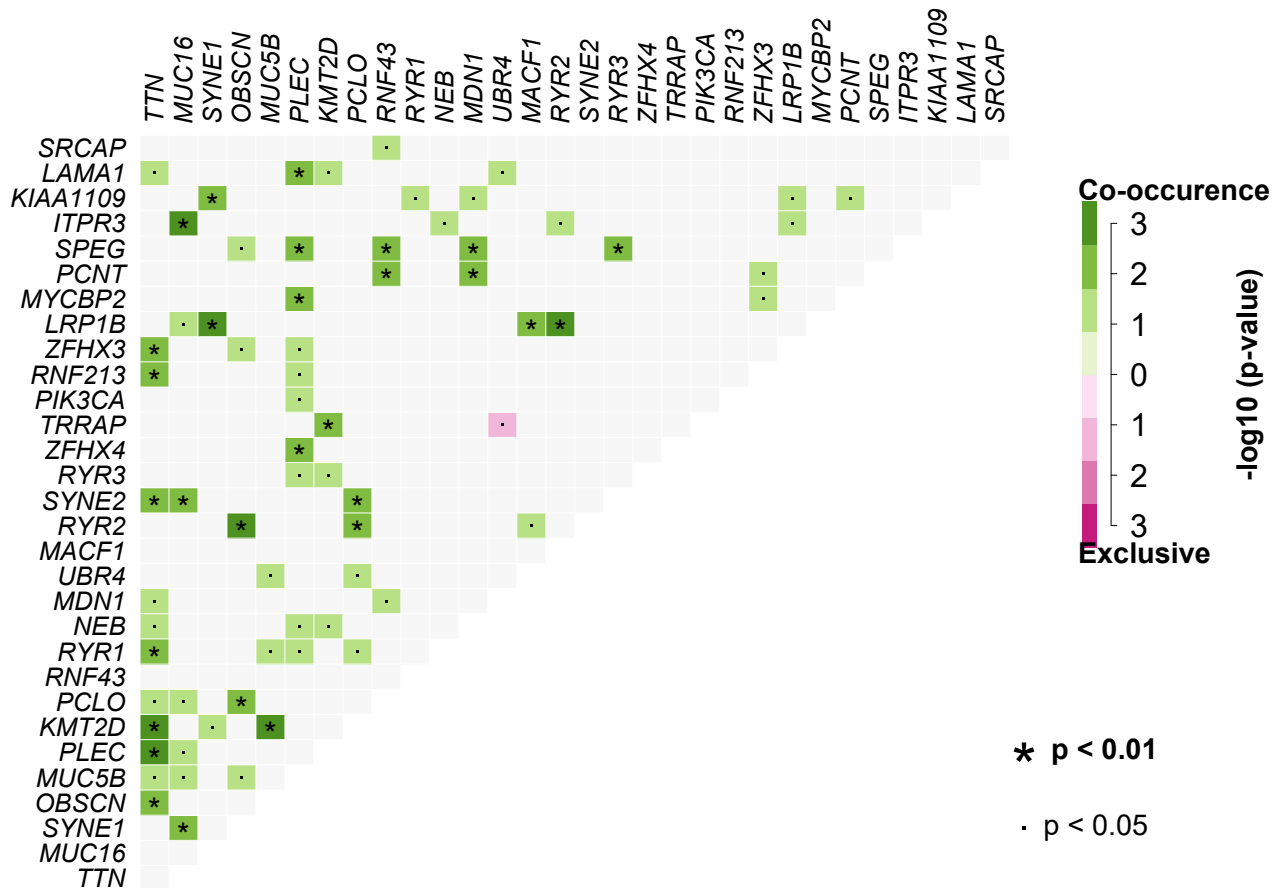

Supplement: Supplementary Materials — Figure S1: the landscape of 25 significantly mutated genes in all the CRC patients. Figure S2: ratio of base conversion and transversion in mutations. Figure S3: the landscape of 25 significantly mutated genes in MSI-H CRC patients. Figure S4: the landscape of 25 significantly mutated genes in MSS CRC patients. Figure S5: somatic interaction analysis among the significantly mutated genes found four genes mutually exclusive with TP53 in MSS samples. Figure S6: somatic interaction analysis in MSI-H samples. Table S1: the list of clinical information of samples in previous seven studies of CRC. Table S2: the list of high-frequency HLA genotypes in Chinese and TCGA. Table S3: the list of SNV derived neoantigens. Table S4: the list of indel-derived neoantigens. [file 2861240.f1.zip › Figure S6.pdf]
