# Supplementary material for: Recurrent Neoantigens in Colorectal Cancer as Potential Immunotherapy Targets
Source: Biomed Res Int. 2020 Jul 17;2020:2861240. doi: 10.1155/2020/2861240 (PMC7383341; doi:10.1155/2020/2861240)
Supplement: Supplementary Materials — Figure S1: the landscape of 25 significantly mutated genes in all the CRC patients. Figure S2: ratio of base conversion and transversion in mutations. Figure S3: the landscape of 25 significantly mutated genes in MSI-H CRC patients. Figure S4: the landscape of 25 significantly mutated genes in MSS CRC patients. Figure S5: somatic interaction analysis among the significantly mutated genes found four genes mutually exclusive with TP53 in MSS samples. Figure S6: somatic interaction analysis in MSI-H samples. Table S1: the list of clinical information of samples in previous seven studies of CRC. Table S2: the list of high-frequency HLA genotypes in Chinese and TCGA. Table S3: the list of SNV derived neoantigens. Table S4: the list of indel-derived neoantigens. [file 2861240.f1.zip › Supplementary Files Descriptions_BMRI_3097961.doc]

## Supplementary Materials

Figure S1: The landscape of 25 significantly mutated genes in all the CRC patients.

Figure S2: Ratio of base conversion and transversion in mutations.

Figure S3: The landscape of 25 significantly mutated genes in MSI-H CRC patients.

Figure S4: The landscape of 25 significantly mutated genes in MSS CRC patients.

Figure S5: Somatic interaction analysis among the significantly mutated genes found four genes mutually exclusive with *TP53 in MSS samples*.

Figure S6: Somatic interaction analysis in MSI-H samples.

Table S1: The list of clinical information of samples in previous seven studies of CRC.

Table S2: The list of high-frequency HLA genotypes in Chinese and TCGA.

Table S3: The list of SNV derived neoantigens.

Table S4: The list of indel derived neoantigens.
